# Development and Validation of a Prognostic Signature Based on the Lysine Crotonylation Regulators in Head and Neck Squamous Cell Carcinoma

**DOI:** 10.1155/2023/4444869

**Published:** 2023-02-13

**Authors:** Linlin Jiang, Xiteng Yin, Hongbo Zhang, Xinyu Zhang, Zichen Cao, Meng Zhou, Wenguang Xu

**Affiliations:** ^1^Department of Medical Oncology, The Second Hospital of Nanjing, Nanjing University of Chinese Medicine, Nanjing, China; ^2^Department of Oral and Maxillofacial Surgery, Nanjing Stomatological Hospital, Medical School of Nanjing University, Nanjing, China; ^3^Central Laboratory of Stomatology, Nanjing Stomatological Hospital, Medical School of Nanjing University, Nanjing, China; ^4^Department of Oral and Maxillofacial Surgery, The Affiliated Stomatological Hospital of Xuzhou Medical University, Xuzhou, China

## Abstract

**Background:**

Lysine crotonylation (Kcr) is a newly identified posttranslational modification type regulated by various enzymes and coenzymes, including lysine crotonyltransferase, lysine decrotonylase, and binding proteins. However, the role of Kcr regulators in head and neck squamous cell carcinoma (HNSCC) remains unknown. The aim of this study was to establish and validate a Kcr-related prognostic signature of HNSCC and to assess the clinical predictive value of this signature.

**Methods:**

The mRNA expression profiles and clinicopathological data from The Cancer Genome Atlas (TCGA) database were downloaded to explore the clinical significance and prognostic value of these regulators in HNSCC. The least absolute shrinkage and selection operator (LASSO) Cox regression model was used to generate the Kcr-related prognostic signature for HNSCC. Subsequently, the GSE65858 dataset from the Gene Expression Omnibus (GEO) database was used to validate the signature. The prognostic value of the signature was evaluated using the Kaplan-Meier survival, receiver operating characteristic (ROC) curve, and univariate and multivariate Cox regression analyses.

**Results:**

We established a nine-gene risk signature associated with the prognosis of HNSCC based on Kcr regulators. High-risk patients demonstrated significantly poorer overall survival (OS) than low-risk patients in the training (TCGA) and validation (GEO) datasets. Then, the time-dependent receiver operating characteristic (ROC) curve analysis showed that the nine-gene risk signature was more accurate for predicting the 5-year OS than other clinical parameters, including age, gender, T stage, N stage, and histologic grade in the TCGA and GEO datasets. Moreover, the Cox regression analysis showed that the constructed risk signature was an independent risk factor for HNSCC.

**Conclusion:**

Our study identified and validated a nine-gene signature for HNSCC based on Kcr regulators. These results might contribute to prognosis stratification and treatment escalation for HNSCC patients.

## 1. Introduction

Head and neck cancer ranks as the seventh most common cancer worldwide in 2018, accounting for 3% of all malignancies [1]. Head and neck squamous cell carcinomas (HNSCC) are the most common malignancies in the head and neck region, developing from the mucosal epithelium in the oral cavity, pharynx, and larynx [2]. Despite considerable therapeutic advances in managing HNSCC, the overall survival (OS) rate of HNSCC patients remained dismal in recent decades [3, 4]. Currently, the TNM (tumor, node, and metastasis) staging system established by the American Joint Committee on Cancer (AJCC) is used to classify HNSCC and determine treatment modalities [5]. However, the TNM stage performance is not ideal, as HNSCC patients with the same TNM stage still differ in clinical outcomes. Numerous efforts have been made to develop an optimal tool for HNSCC risk stratification and prognosis prediction, but no consensus has been achieved. With the rapid development of next-generation sequencing technologies, integrating prognostic gene signatures and traditional clinicopathologic factors has shown an excellent advantage for HNSCC prognosis prediction [2]. Therefore, developing accurate and robust prognostic signatures is critical to help oncologists optimize therapeutic strategies for HNSCC.

Posttranslational modification of proteins occurs in all living organisms and has been increasingly recognized to play a vital role in various biological processes, including gene expression regulation, cell growth, embryo development, and metabolism [6, 7]. As an amphipathic residue with a hydrophobic side chain, lysine acylation neutralizes the positive charge of the amino group, changing protein conformation. Lysine acylations include acetylation, succinylation, malonylation, glutarylation, crotonylation, and *β*-hydroxybutyrylation [8]. Lysine crotonylation (Kcr) is a newly discovered posttranslational modification identified on histones and nonhistones [9].

Lysine crotonylation is a dynamic reversible process composed of crotonyltransferase complex (writers), decrotonylases (erasers), and binding proteins (readers). Crotonyltransferases, colloquially termed writers, promote the covalent modification of Kcr proteins, including CBP/p300, MOF, Gcn5, Esa1, and PCAF [10–14]. In contrast, decrotonylases, colloquially termed erasers, remove the covalent modification of Kcr proteins, including SIRT1, SIRT2, SIRT3, HDAC1, HDAC2, HDAC3, and HDAC8 [15–17]. Readers are responsible for “reading” Kcr information and participating in the recruitment of downstream readers, such as TAF1, AF9, YEATS2, Taf14, MOZ, and DPF2 [18–21]. Previously, a quantitative proteomics study revealed that p300-targeted Kcr substrates were potentially linked to cancer and might act as carcinogenic factors to promote tumor progress [22]. Additionally, p300 upregulates HNRNPA1 expression by lysine crotonylation to promote the proliferation, invasion, and migration of HeLa cells *in vitro* [23]. In hepatocellular carcinoma, lysine crotonylation expression is associated with TNM stages. However, no correlation was found between lysine crotonylation expression and the prognosis of patients [24]. Currently, the underlying processes and molecular alterations of lysine crotonylation in HNSCC remain unknown, especially regarding its prognostic potential.

In the present study, we systematically analyzed the expression patterns of 18 widely reported Kcr regulators in 491 HNSCC patients with RNA sequencing (RNA-seq) data from The Cancer Genome Atlas (TCGA) database. We explored their potential roles in HNSCC oncogenesis and progression. Finally, a nine-gene risk signature was built to stratify HNSCC prognoses based on TCGA cohort. This robust prognostic signature was successfully validated in an independent external cohort from the Gene Expression Omnibus (GEO) database.

## 2. Materials and Methods

### 2.1. Data Acquisition

The RNA-seq transcriptome data normalized by the Expectation-Maximization (RSEM) approach of HNSCC and normal control samples and the corresponding clinical data of HNSCC patients were retrieved from TCGA (https://portal.gdc.cancer.gov/). The RNA expression profiles of HNSCC samples and corresponding clinical data in the GSE65858 dataset were acquired from the GEO database. The clinicopathological information for TCGA and GEO datasets is summarized in Supplementary Tables S1 and S2.

### 2.2. Selection of Kcr Regulators

First, we manually collected a list of Kcr regulators from the literature [25–27]. The selection criteria for inclusion of Kcr regulators were (1) corresponding regulatory mechanisms validated *in vivo* or *in vitro*, (2) regulators implicated in various physiological and pathological processes, and (3) available RNA expression data in TCGA and GEO datasets. Finally, we collected 18 Kcr regulators. The expression matrix of these genes and the clinicopathological data of samples were extracted and used for subsequent bioinformatics analysis. These genes and corresponding aliases are listed in Supplementary Table S3.

### 2.3. Differential Expression Analysis of Kcr Regulators

The “limma” R package was used to screen differentially expressed Kcr regulators in 502 tumor and 44 normal control samples. An adjusted *p* < 0.05 and |log2 fFold cChange (FC)| > 1 were set as the cutoff threshold. Heatmap and violin plots were used to visualize the differential expression of the 18 genes in 502 tumor and 44 normal samples.

Next, the differential expressions of the 18 genes in tumor samples with different clinicopathological parameters were determined using the “limma” R package and visualized in heatmaps using the “pheatmap” package. Differentially expressed genes significantly correlated with clinicopathological parameters were further visualized in bar plots.

### 2.4. Protein-Protein Interaction (PPI) Network Construction and Correlation Analysis

The STRING database was used to analyze the PPI network among Kcr regulators [28]. The Pearson correlation analysis was used to explore the associations among different Kcr regulators.

### 2.5. Prognostic Signature Construction and Validation

The univariate Cox regression analysis was performed to identify Kcr regulators harboring prognostic value in TCGA database. Genes with hazard ratio (HR) < 1 have better OS, while genes with HR > 1 have worse OS. Considering the limited number of prognostic genes, all genes were included in the subsequent analysis to develop a risk signature with the least absolute shrinkage and selection operator (LASSO) Cox regression algorithm. The prognostic gene signature is represented by the following: risk score = (coefficient of mRNA1 × expression of mRNA1) + (coefficient of mRNA2 × expression of mRNA2) + ⋯ + (coefficient of mRNAn × expression mRNAn). This formula was used to calculate a risk score for each patient in the training (TCGA) and validation (GEO) cohorts. The cohort was stratified into high- and low-risk groups based on the median value of the risk scores.

Before validation, the “sva” R package was used to conduct batch normalization of the expression data between TCGA and GEO datasets. The “survminer” package was used to draw the Kaplan-Meier survival curve. The “survivalROC” package was used to investigate the time-dependent prognostic value of the gene signature and clinicopathological variables.

### 2.6. Gene Set Enrichment Analysis (GSEA)

The GSEA was performed to identify enriched Gene Ontology (GO) terms and reveal potential underlying Kyoto Encyclopedia of Genes and Genomes (KEGG) pathways of the gene signature. A *p* < 0.05 and a false discovery rate *q* < 0.25 were considered statistically significant.

### 2.7. Statistical Analysis

All statistical analyses were conducted using R software (v. 3.5.2). The Wilcoxon test was used to compare the expression of genes among different groups. The *χ*^2^ test was conducted to compare the distribution of clinicopathological variables between high- and low-risk groups. The Kaplan-Meier analysis was conducted to compare the OS between high- and low-risk groups using the log-rank test. Univariate and multivariate Cox regression analyses were used to determine independent prognostic factors for the training and validation cohorts. The ROC curve was used to evaluate the accuracy of the constructed gene signature. All statistical tests were performed using a two-sided *p* < 0.05 as the significant threshold.

## 3. Results

### 3.1. The Expression of Kcr Regulators Is Correlated with HNSCC Tumorigenesis and Progression

**Figure 1:**
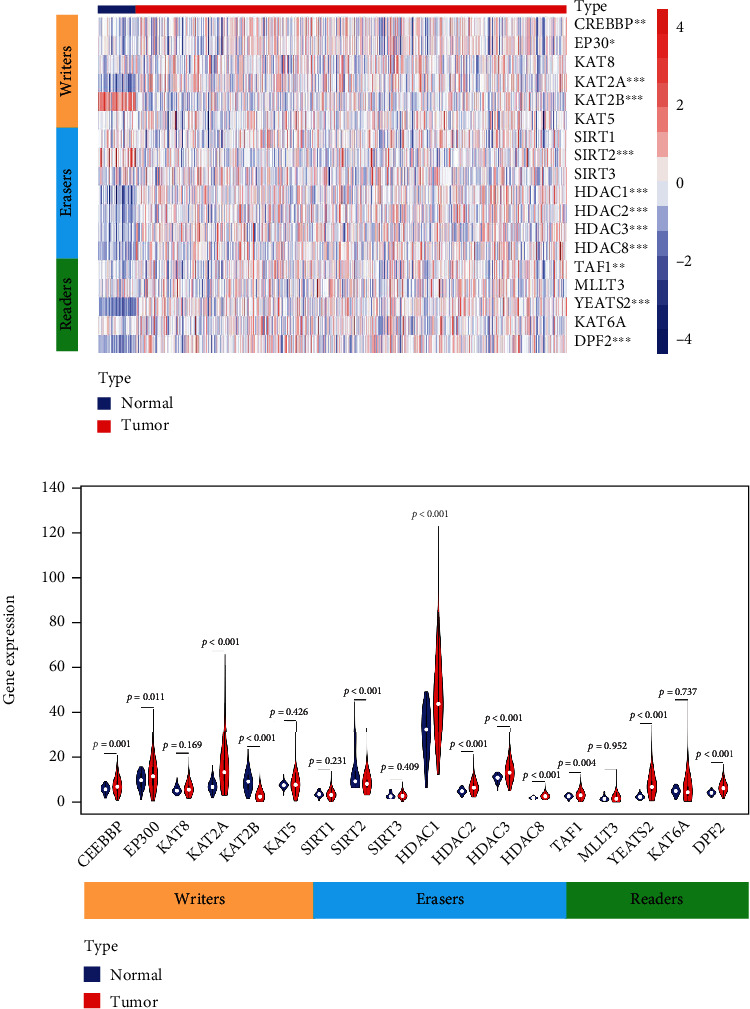
Expression levels of lysine crotonylation regulators between cancerous and adjacent normal samples in TCGA HNSCC cohort. (a) Heatmap with expression levels of lysine crotonylation regulators in each clinical sample. (b) Violin plot of significantly differentially expressed lysine crotonylation regulators between cancerous and adjacent normal samples.

**Figure 2:**
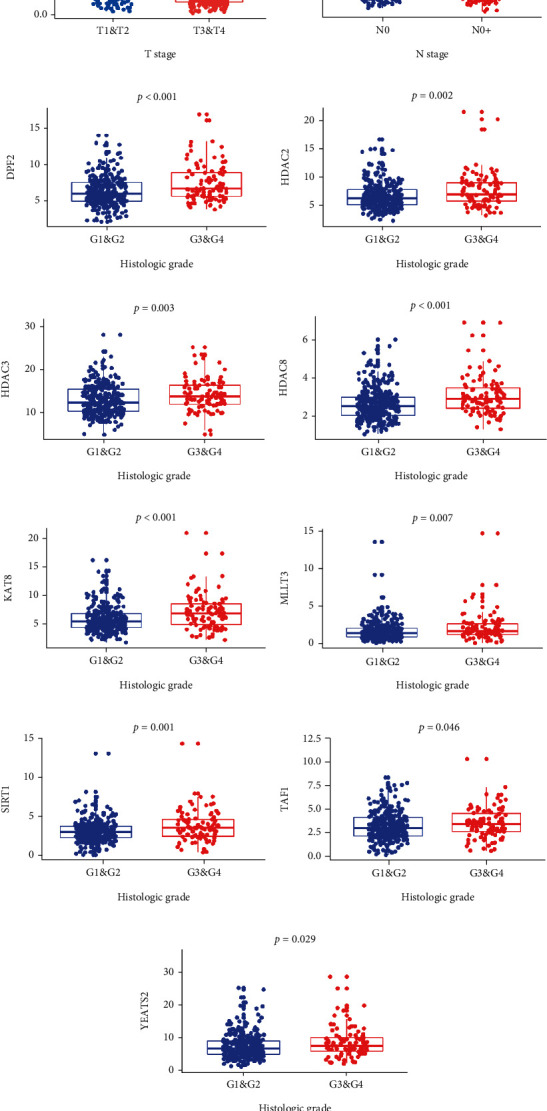
Significantly differentially expressed lysine crotonylation regulators in HNSCC with different clinicopathological features. (a) KAT2B expression in HNSCC patients with early T stage (T1 and T2) and advanced T stage (T3 and T4). (b) HDAC2 expression in HNSCC patients with and without lymph node metastasis. (c–k) Expression levels of DPF2, HDAC2, HDAC3, HDAC8, KAT8, MLLT3, SIRT1, TAF1, and YEATS2 in HNSCC patients with different histologic grades, respectively.

First, we visualized the expression pattern of Kcr regulators between HNSCC and normal controls using a heatmap and violin plot (Figure 1). We found that CREBBP (*p* = 0.001), EP300 (*p* = 0.011), KAT2A (*p* < 0.001), HDAC1 (*p* < 0.001), HDAC2 (*p* < 0.001), HDAC3 (*p* < 0.001), HDAC8 (*p* < 0.001), TAF1 (*p* = 0.004), and YEATS2 (*p* < 0.001) were significantly upregulated in HNSCC samples compared to normal samples, while KAT2B (*p* < 0.001) and SIRT2 (*p* < 0.001) were remarkably downregulated in HNSCC samples (Figure 1(b)). Next, we systematically investigated the relationships between each Kcr regulator and the clinicopathologic features of HNSCC patients, including tumor stage, presence of lymph node metastasis, and histologic grade. The expressions of each Kcr regulator stratified by tumor stage, presence of lymph node metastasis, and histologic grade are presented as heatmaps (Supplementary Figure 1). Specifically, KAT2B was downregulated in the advanced T stage compared to the early T stage (*p* = 0.0016). Meanwhile, HDAC2 was upregulated in HNSCC patients with lymph node metastasis compared to those without lymph node metastasis (*p* = 0.035). Additionally, most Kcr regulators, including DPF2, HDAC2, HDAC3, HDAC8, KAT8, MLLT3, SIRT1, TAF1, and YEATS2, were significantly upregulated in HNSCC patients with higher histologic grade (Figure 2).

### 3.2. Interaction and Correlation among Kcr Regulators

**Figure 3:**
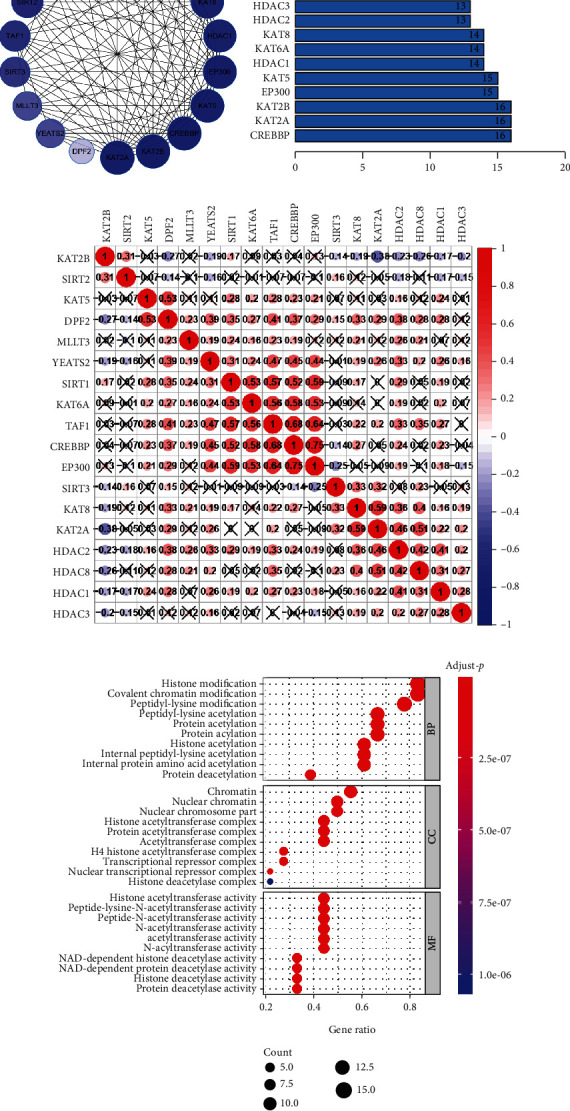
Interaction among lysine crotonylation regulators and functional annotation. (a) Protein-protein interactions between the 18 lysine crotonylation regulators. The color intensity in each node is proportional to the degree of connectivity in the network. (b) Degree of connectivity of each gene in the network. (c) Spearman correlation analysis of the 18 lysine crotonylation regulators in HNSCC. (d) Functional annotation of lysine crotonylation regulators by GO analysis.

The PPIs among the 18 Kcr regulators are presented in Figure 3(a). According to the degree of connectivity of each gene in the interaction network, the “writers” CREBBP, KAT2A, and KAT2B were hub genes (Figure 3(b)). The correlation analysis revealed that CREBBP was most relevant with EP300 (*r*^2^ = 0.75) among all interactions of crotonylation regulators. Interestingly, several independent interaction groups were detected for “writers,” “readers,” and “erasers,” indicating the diverse functional pathways of different regulators. More specifically, the expressions of CREBBP, EP300, TAF1, KAT6A, YEATS2, and SIRT1 and KAT2A, KAT8, HDAC1, HDAC2, HDAC3, and HDAC8 were significantly correlated with each other in HNSCC (Figure 3(c)). To explore the potential functions of Kcr regulators, we conducted a GO analysis. These regulators were mainly involved in some biological processes, including histone modification, covalent chromatin modification, and peptidyl-lysine modification, and some molecular functions, including histone acetyltransferase, peptide-lysine-N-acetyltransferase, and peptide N-acetyltransferase activities (Figure 3(d)).

### 3.3. Development and Validation of a Prognostic Signature Based on Kcr Regulators

To investigate the prognostic value of Kcr regulators, we first performed a univariate Cox regression on the expression levels in TCGA dataset. The results revealed that CREBBP, KAT2B, and KAT6A were significantly correlated with OS (*p* < 0.05) and were protective genes (HR < 1) (Supplementary Figure 2A). Then, we applied the LASSO Cox regression algorithm to better predict the clinical outcomes of HNSCC patients using Kcr regulators. Finally, nine genes were screened out to construct the risk signature based on the minimum criteria, and the coefficients from the LASSO algorithm were used to establish the risk signature for both the training (TCGA) and validation (GEO) datasets (Supplementary Figures 2B and C): Risk score = (0.001876 × expression value of EP300) + (−0.049122 × expression value of KAT8) + (−0.001630 × expression value of KAT2A) + (−0.047159 × expression value of KAT2B) + (0.029484 × expression value of HDAC2) + (0.024618 × expression value of HDAC3) + (0.061997 × expression value of MLLT3 + (0.008895 × expression value of YEATS2) + (−0.051189 × expression value of KAT6A).

**Figure 4:**
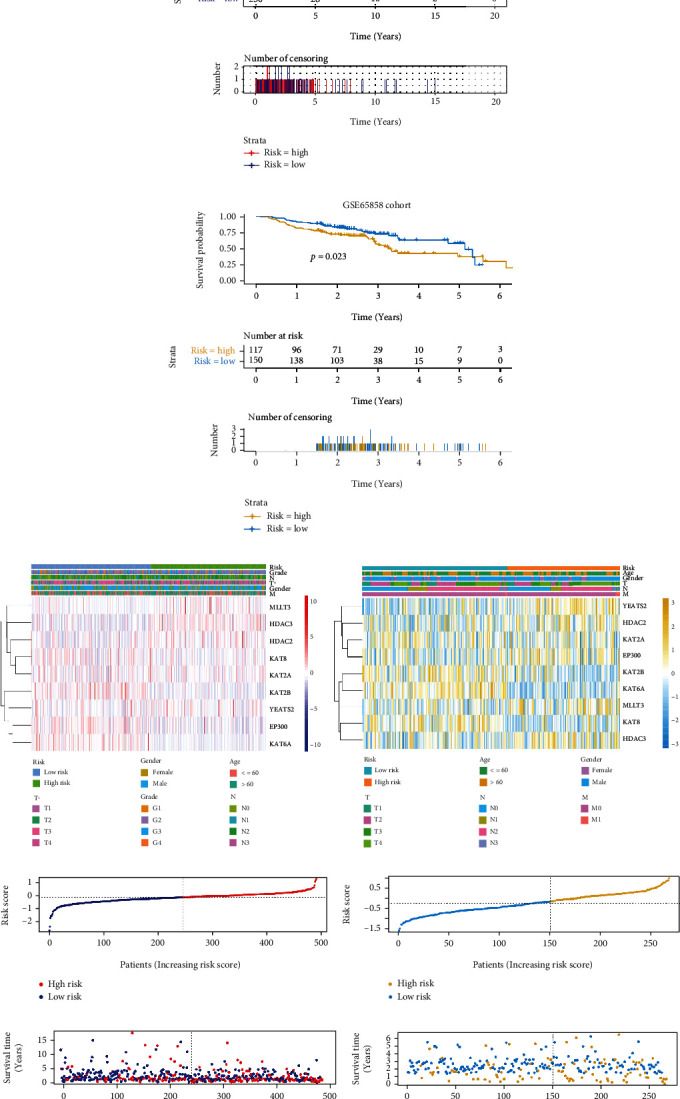
Risk signature with nine lysine crotonylation regulators in the training and validation cohorts. (a, b) Kaplan-Meier curves for the OS of patients stratified by the nine-gene prognostic signature into high- and low-risk groups in the training and validation cohorts. (c, d) Heatmap of the nine prognostic genes that were differentially expressed between the high- and low-risk groups in the training and validation cohorts. (e, f) Risk scores plotted in ascending order and marked as low or high risk for all patients in the training and validation cohorts. (g, h) Survival status distribution of HNSCC patients in the training and validation cohorts.

Based on the nine-gene risk signature, all 491 HNSCC patients in the training dataset were divided into high- and low-risk groups according to the median risk score. Significant differences were observed between the two groups (*p* < 0.0001) (Figure 4(a)). To test the robustness and clinical practice of the nine-gene risk signature, 267 HNSCC patients from another independent external dataset (GEO) were also divided into high- and low-risk groups according to the same risk score cutoff point obtained from the training dataset. The nine-gene risk signature could also effectively stratify patients into low- and high-risk groups with significantly different OS in the GEO-HNSCC dataset (*p* < 0.05) (Figure 4(b)). Additionally, the expressions of the nine prognostic genes in high- and low-risk groups in TCGA and GEO datasets are presented in Figures 4(c) and 4(d).Notably, we found a significant difference between high- and low-risk groups for T stages (*p* < 0.05) in TCGA dataset. Moreover, all patients in the training and validation cohorts were ranked from left to right according to the risk score (Figures 4(e) and 4(f)). Accordingly, the distribution of the survival status of each patient in the training and validation cohorts is presented in Figures 4(g) and 4(h).

### 3.4. Performance Comparison by Time-Dependent ROC Curve Analysis

**Figure 5:**
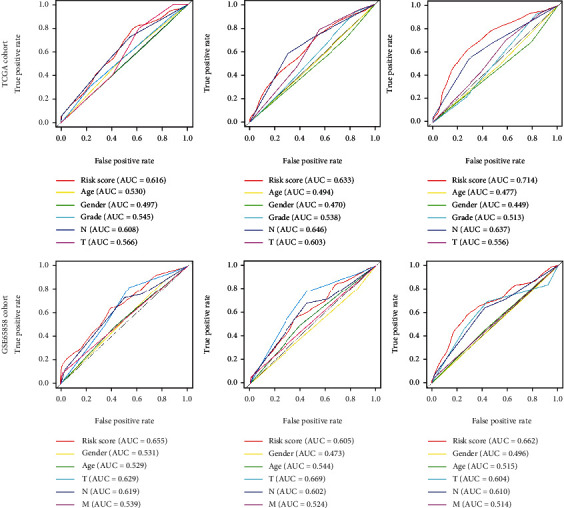
Time-dependent ROC analysis of the sensitivity and specificity for survival prediction at 1, 3, and 5 years in the training and validation cohorts. (a–c) Time-dependent ROC curves for survival prediction of the nine-gene prognostic signature compared to clinicopathologic variables at 1, 3, and 5 years in the training cohort. (d–f) Time-dependent ROC curves for survival prediction of the nine-gene prognostic signature compared to clinicopathologic variables at 1, 3, and 5 years in the validation cohort.

We performed the time-dependent ROC curve analysis to compare the prediction performance of the nine-gene risk signature with other clinicopathologic variables, including age, gender, T stage, N stage, and histologic grade in TCGA and GSE65858 cohorts. In TCGA cohort, the risk signature could predict well the 1-, 3-, and 5-year OS rates of HNSCC patients (Figures 5(a)–5(c)). Particularly, the predictive efficiency of the risk signature at 5 years was better than the T stage, N stage, histologic grade, age, and gender (Figure 5(c)). The validation in the GSE65858 cohort further demonstrated a moderate sensitivity and specificity of the risk signature at 1, 3, and 5 years (Figures 5(d)–5(f)).

### 3.5. Independent Prognostic Role of the Gene Signature

**Figure 6:**
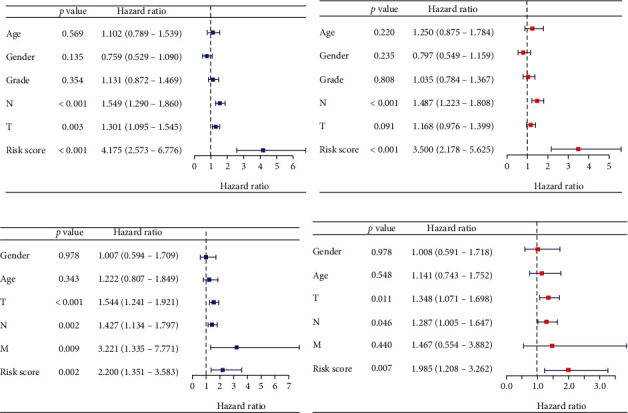
Forrest plot for the univariate and multivariate Cox regression analyses in HNSCC patients in the training and validation cohorts. (a, b) Forrest plots of the univariate and multivariate Cox regression analyses in HNSCC patients in TCGA cohort. (c, d) Forrest plots of the univariate and multivariate Cox regression analyses in HNSCC patients in the GSE65858 cohort.

To determine whether the risk signature was an independent prognostic indicator for HNSCC, univariate and multivariate Cox regression analyses were performed in TCGA and GSE65858 cohorts. In the training cohort, the univariate analysis revealed that the T stage (*p* = 0.003, HR = 1.301, 95% confidence interval (CI) = 1.095 − 1.545), N stage (*p* < 0.001, HR = 1.549, 95%CI = 1.290 − 1.860), and risk signature (*p* < 0.001, HR = 4.175, 95%CI = 2.573 − 6.776) were significantly correlated with OS (Figure 6(a)). The multivariate analysis further identified N stage (*p* < 0.001, HR = 1.487, 95%CI = 1.223 − 1.808) and risk signature (*p* < 0.001, HR = 3.500, 95%CI = 2.178 − 5.625) as independent prognostic factors (Figure 6(b)). Next, we performed univariate and multivariate Cox regression analyses for HNSCC patients in the GSE65858 cohort to validate the prognostic value of the risk signature. The risk score (*p* = 0.002, HR = 2.200, 95%CI = 1.351 − 3.583), T stage (*p* < 0.001, HR = 1.544, 95%CI = 1.241 − 1.921), N stage (*p* = 0.002, HR = 1.427, 95%CI = 1.134 − 1.797), and M stage (*p* = 0.009, HR = 3.221, 95%CI = 1.335 − 7.771) were significantly associated with the OS in the univariate analysis (Figure 6(c)). In the multivariate analysis, the risk score (*p* = 0.007, HR = 1.985, 95%CI = 1.208 − 3.262), T stage (*p* = 0.011, HR = 1.348, 95%CI = 1.071 − 1.698), and N stage (*p* = 0.046, HR = 1.287, 95%CI = 1.005 − 1.647) remained independent prognostic factors for HNSCC patients (Figure 6(d)). Altogether, these results indicated that the risk signature derived from the Kcr regulators was an independent prognostic indicator for HNSCC.

### 3.6. Identification of the Involved Biological Processes and KEGG Pathways by GSEA

**Figure 7:**
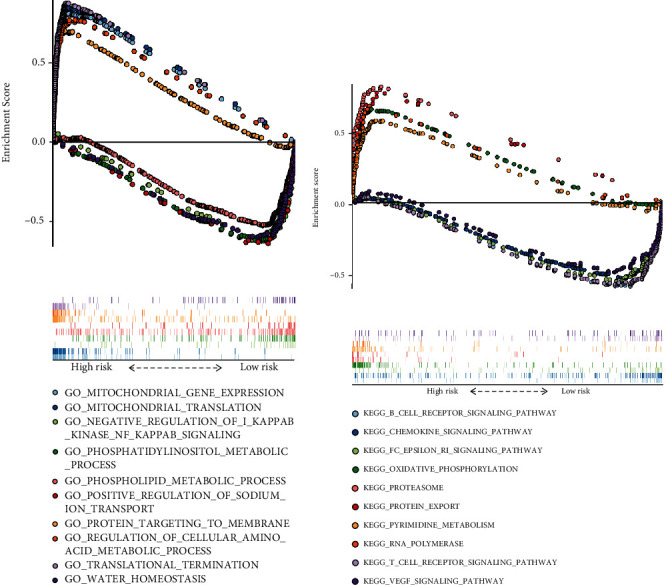
Biological processes and KEGG pathways enriched by GSEA. (a) Representative biological processes enriched and (b) KEGG pathways in high- and low-risk HNSCC patients.

Further, we performed GSEA to determine the biological processes and KEGG pathways enriched in high- and low-risk HNSCC patients. Five representative biological processes—mitochondrial gene expression, mitochondrial translation, negative regulation of I*κ*B kinase NF-*κ*B signaling, phosphatidylinositol metabolic process, and phospholipid metabolic process—were enriched in high-risk patients. In contrast, positive regulation of sodium ion transport, protein targeting to membrane, regulation of cellular amino acid metabolic processes, translation termination, and water homeostasis were enriched in low-risk patients (Figure 7(a)). Regarding KEGG pathways, the B cell receptor signaling pathway, chemokine signaling pathway, FC epsilon RI signaling pathway, oxidative phosphorylation, and proteasome were enriched in high-risk patients. On the other hand, protein export, pyrimidine metabolism, RNA polymerase, T cell receptor signaling pathway, and VEGF signaling pathway were enriched in low-risk patients (Figure 7(b)).

## 4. Discussion

In our study, we identified a robust nine-gene risk signature for HNSCC patients in TCGA database using LASSO and univariate Cox regression analyses. With further validation in the GSE65858 dataset, the risk signature was shown to be an independent prognostic indicator for HNSCC patients. Risk scores derived from this signature could effectively stratify HNSCC patients into low- and high-risk groups. Importantly, the time-dependent ROC curve analysis revealed that the nine-gene risk signature was more accurate for predicting the 5-year OS than other clinical parameters, including age, gender, T stage, N stage, and histologic grade. Therefore, compared to the traditional staging system, the constructed risk signature showed an advantage in predicting the prognosis of HNSCC patients, which might contribute to prognosis stratification and treatment escalation for HNSCC patients.

As a newly identified posttranslational modification, Kcr is specifically enriched on active gene promoters or potential enhancers in mammalian cell genomes [29]. Since it was reported in 2011, emerging evidence has demonstrated that Kcr is involved in multiple physiological and pathological processes, including spermatogenesis, neuropsychiatric disease, tissue injury, inflammation, and tumorigenesis [25]. Previously, few studies have focused on the relationship between Kcr and cancer. Wan et al. found that the global expression of Kcr was downregulated in liver, gastric, and renal carcinomas, while it was upregulated in thyroid, esophagus, colon, pancreas, and lung malignancies by immunohistochemical staining [24]. Particularly, Kcr expression was associated with the TNM stage in hepatocellular carcinoma. However, no prognostic significance of lysine crotonylation was found in hepatocellular carcinoma.

In this study, the clinical significance of Kcr in HNSCC was initially investigated. The differential analysis revealed that most Kcr regulators were aberrantly expressed in HNSCC. Specifically, CREBBP, EP300, KAT2A, HDAC1, HDAC2, HDAC3, HDAC8, TAF1, and YEATS2 were significantly upregulated, while KAT2B and SIRT2 were downregulated in HNSCC samples. Besides, various Kcr regulators, including KAT2B, DPF2, HDAC2, HDAC3, HDAC8, KAT8, MLLT3, SIRT1, TAF1, and YEATS2, were correlated with T stage, lymph node metastasis, and histologic grade. These results indicated that Kcr regulators might contribute to cancer progression in HNSCC.

Based on the interactions between Kcr regulators, CREBBP seemed to be the most relevant regulator with EP300, consistent with previous studies [30, 31]. CREBBP and EP300 are widely recognized histone acetyltransferases and transcriptional coactivators that share approximately 60% homology and play vital roles in various cellular activities such as cell growth, differentiation, DNA repair, and apoptosis [32–34]. Notably, CREBBP was previously reported as a novel tumor suppressor, and CREBBP dysfunction was correlated with carcinogenesis and progression in several human malignancies [35, 36]. Similarly, our findings also revealed that high CREBBP expression was associated with a favorable HNSCC prognosis, indicating that CREBBP might play a tumor-suppressive role in HNSCC. However, Hu et al. demonstrated that CREBBP acted as an oncogene and predicted a poor prognosis in ovarian cancer [37]. These differences might be due to tumor heterogeneity among different malignancies.

Furthermore, KAT2B was also identified as a hub gene in the PPI network. Previous studies have revealed the relationship between KAT2B and tumor occurrence and development. Bharathy et al. found that KAT2B was overexpressed in primary alveolar rhabdomyosarcoma, and its acetylation activated the PAX3-FOXO1 pathway and promoted carcinogenesis [38]. Malatesta et al. demonstrated that KAT2B was an important factor in the Hedgehog signaling pathway, and its downregulation in medulloblastoma and glioblastoma cells contributed to decreased proliferation and increased apoptosis [39]. Conversely, KAT2B plays an oncogenic role in multiple types of cancer, such as gastric, liver, and cervical cancers. Moreover, KAT2B suppressed the tumorigenicity of gastric cancer *in vitro* and *in vivo* and was correlated with aggressive clinical features [40]. KAT2B was downregulated in hepatocellular carcinoma tissues and significantly associated with a favorable prognosis for patients [41]. Similarly, KAT2B was significantly downregulated in cervical cancer tissues, and its low expression was closely associated with a poor prognosis [41]. Here, KAT2B was downregulated in HNSCC samples and was negatively correlated with the T stage. The survival analysis also validated KAT2B as a favorable prognostic biomarker for HNSCC.

In our study, we identified a robust nine-gene risk signature for HNSCC patients in TCGA database using LASSO and univariate Cox regression analyses. With further validation in the GSE65858 dataset, the risk signature was shown to be an independent prognostic indicator for HNSCC patients. Risk scores derived from this signature could effectively stratify HNSCC patients into low- and high-risk groups. Importantly, the time-dependent ROC curve analysis revealed that the nine-gene risk signature was more accurate for predicting the 5-year OS than other clinical parameters, including age, gender, T stage, N stage, and histologic grade. Therefore, compared to the traditional staging system, the constructed risk signature showed an advantage in predicting the prognosis of HNSCC patients, which might contribute to prognosis stratification and treatment escalation for HNSCC patients.

However, our current study also has some limitations. Given the retrospective nature of this study, further validation in prospective and multicenter clinical trials is essential to validate the accuracy and efficiency of the constructed signature. Moreover, further experimental studies are needed to verify the role of Kcr and elucidate the underlying mechanisms of the signature in HNSCC. Additionally, due to the few studies investigating the role of Kcr in tumors, the information on Kcr regulators was manually extracted from the literature. Thus, some latent and unidentified Kcr regulators might be omitted in the gene sets.

## 5. Conclusion

In summary, we systematically demonstrated the expression profiles and clinical significance of Kcr regulators in HNSCC patients. We established a nine-gene prognostic signature based on the Kcr regulators and validated it in an external HNSCC cohort. The constructed risk signature was an independent prognostic factor for HNSCC patients, which could effectively predict the survival of HNSCC patients and facilitate clinical decision-making for oncologists. Multicenter and prospective studies with large sample sizes are needed to further validate the clinical practicality and accuracy of the risk signature.

## Data Availability

The data that support the findings of this study are available in TCGA database at https://www.cancer.gov/ and GEO database at https://www.ncbi.nlm.nih.gov/geo/ (reference number: GSE65858).

## Supplementary Materials

Supplementary Materials Supplementary Table S1: clinicopathologic characteristics of HNSCC patients in TCGA cohort. Supplementary Table S2: clinicopathologic characteristics of HNSCC patients in the GEO cohort. Supplementary Table S3: gene symbols of lysine crotonylation regulators. Supplementary Figure 1: expression of lysine crotonylation regulators in HNSCC with different clinicopathological features. Expression levels of 18 lysine crotonylation regulators in HNSCC with (A) different T stage groups, (B) with or without lymph node metastasis, and (C) different histologic grades. ^∗^*p* < 0.05, ^∗∗^*p* < 0.01, and ^∗∗∗^*p* < 0.001. Supplementary Figure 2: construction of prognostic signatures based on the crotonylation regulators in TCGA-HNSCC. (A) Univariate Cox regression of lysine crotonylation regulators on the prognosis of HNSCC patients in TCGA dataset. (B) The coefficient profile was plotted against the log (lambda) sequence. Selection of the optimal parameter (lambda) in the LASSO model for HNSCC. (C) LASSO coefficient profiles of the 18 crotonylation regulators in HNSCC.
