# Supplementary material for: Development and Validation of a Prognostic Signature Based on the Lysine Crotonylation Regulators in Head and Neck Squamous Cell Carcinoma
Source: Biomed Res Int. 2023 Feb 13;2023:4444869. doi: 10.1155/2023/4444869 (PMC9940974; doi:10.1155/2023/4444869)
Supplement: Supplementary Materials — Supplementary Table S1: clinicopathologic characteristics of HNSCC patients in TCGA cohort. Supplementary Table S2: clinicopathologic characteristics of HNSCC patients in the GEO cohort. Supplementary Table S3: gene symbols of lysine crotonylation regulators. Supplementary Figure 1: expression of lysine crotonylation regulators in HNSCC with different clinicopathological features. Expression levels of 18 lysine crotonylation regulators in HNSCC with (A) different T stage groups, (B) with or without lymph node metastasis, and (C) different histologic grades. ∗p < 0.05, ∗∗p < 0.01, and ∗∗∗p < 0.001. Supplementary Figure 2: construction of prognostic signatures based on the crotonylation regulators in TCGA-HNSCC. (A) Univariate Cox regression of lysine crotonylation regulators on the prognosis of HNSCC patients in TCGA dataset. (B) The coefficient profile was plotted against the log (lambda) sequence. Selection of the optimal parameter (lambda) in the LASSO model for HNSCC. (C) LASSO coefficient profiles of the 18 crotonylation regulators in HNSCC. [file 4444869.f1.docx]

**Supporting Information**

**Supplementary Table S1. Clinicopathologic characteristics of HNSCC patients in the TCGA cohort.**

| **Characteristics** | **TCGA cohort, N=491** | |
| --- | --- | --- |
| **Age, years** | **Median** | **Range** |
|  | **61.0** | **19.0-88.0** |
|  | **Number** | **Percentage, %** |
| Gender |  |  |
| Male | 361 | 73.5 |
| Female | 130 | 26.5 |
| Tumor Site |  |  |
| Oral Tongue | 124 | 25.3 |
| Larynx | 110 | 22.4 |
| Base of tongue | 23 | 4.7 |
| Buccal Mucosa | 21 | 4.3 |
| Alveolar Ridge | 18 | 3.7 |
| Tonsil | 39 | 7.9 |
| Floor of mouth | 59 | 12.0 |
| Hard Palate | 6 | 1.2 |
| Oropharynx | 8 | 1.6 |
| Hypopharynx | 10 | 2.0 |
| Oral Cavity | 70 | 14.3 |
| Lip | 3 | 0.6 |
| G category |  |  |
| G1 | 60 | 12.2 |
| G2 | 293 | 59.7 |
| G3 | 117 | 23.8 |
| G4 | 2 | 0.4 |
| GX | 16 | 3.3 |
| NA | 3 | 0.6 |
| UICC stage |  |  |
| I | 25 | 5.1 |
| II | 69 | 14.1 |
| III | 78 | 15.9 |
| IVA | 239 | 48.7 |
| IVB | 11 | 2.2 |
| IVC | 1 | 0.2 |
| Discrepancy | 4 | 0.8 |
| NA | 64 | 13.0 |
| T category |  |  |
| T0 | 1 | 0.2 |
| T1 | 44 | 9.0 |
| T2 | 129 | 26.3 |
| T3 | 96 | 19.6 |
| T4 | 11 | 2.2 |
| T4a | 151 | 30.8 |
| T4b | 4 | 0.8 |
| TX | 33 | 6.7 |
| NA | 22 | 4.5 |
| N category |  |  |
| N0 | 167 | 34.0 |
| N1 | 65 | 13.2 |
| N2 | 12 | 2.4 |
| N2a | 7 | 1.4 |
| N2b | 96 | 19.6 |
| N2c | 44 | 9.0 |
| N3 | 7 | 1.4 |
| NX | 69 | 14.1 |
| NA | 24 | 4.9 |
| Distant metastasis |  |  |
| M0 | 181 | 36.9 |
| M1 | 1 | 0.2 |
| MX | 60 | 12.2 |
| NA | 249 | 50.7 |

UICC, Union for International Cancer Control; NA, not available.

**Supplementary Table S2. Clinicopathologic characteristics of HNSCC patients in the GEO cohort.**

| **Characteristics** |  | **GEO cohort, N=267** |
| --- | --- | --- |
|  | **Median** | **Range** |
| **Age, years** | **58.4** | **35.3-87.4** |
|  | **Number** | **Percentage, %** |
| Gender | | |
| Male | 220 | 82.4 |
| Female | 47 | 17.6 |
| Tumor site | | |
| Cavum Oris | 83 | 31.0 |
| Hypopharynx | 32 | 12.0 |
| Larynx | 48 | 18.0 |
| Oropharynx | 100 | 37.5 |
| NA | 4 | 1.5 |
| UICC stage | | |
| I | 17 | 6.4 |
| II | 37 | 13.9 |
| III | 37 | 13.9 |
| IVA | 154 | 57.7 |
| IVB | 16 | 6.0 |
| IVC | 6 | 2.2 |
| T category | | |
| T1 | 34 | 12.7 |
| T2 | 80 | 30.0 |
| T3 | 57 | 21.3 |
| T4a | 89 | 33.3 |
| T4b | 7 | 2.6 |
| N category | | |
| N0 | 93 | 34.8 |
| N1 | 32 | 12.0 |
| N2a | 11 | 4.1 |
| N2b | 65 | 24.3 |
| N2c | 54 | 20.2 |
| N3 | 12 | 4.5 |
| Distant metastasis | | |
| M0 | 261 | 97.8 |
| M1 | 6 | 2.2 |

UICC, Union for International Cancer Control; NA, not available.

**Supplementary Table S3. Gene symbols of lysine crotonylation regulators.**

| **Official gene symbol** | **Description** | **Aliases** |
| --- | --- | --- |
| CREBBP | CREB binding protein | CBP, KAT3A, MKHK1, RSTS, RSTS1 |
| EP300 | E1A binding protein p300 | KAT3B, MKHK2, RSTS2, p300 |
| KAT8 | lysine acetyltransferase 8 | LIGOWS, MOF, MYST1, ZC2HC8, hMOF |
| KAT2A | lysine acetyltransferase 2A | GCN5, GCN5L2, PCAF-b, hGCN5 |
| KAT2B | lysine acetyltransferase 2B | CAF, P/CAF, PCAF |
| KAT5 | lysine acetyltransferase 5 | ESA1, HTATIP, HTATIP1, PLIP, TIP, TIP60, ZC2HC5, cPLA2 |
| SIRT1 | sirtuin 1 | SIR2, SIR2L1, SIR2alpha |
| SIRT2 | sirtuin 2 | SIR2, SIR2L, SIR2L2 |
| SIRT3 | sirtuin 3 | SIR2L3 |
| HDAC1 | histone deacetylase 1 | GON-10, HD1, KDAC1, RPD3, RPD3L1 |
| HDAC2 | histone deacetylase 2 | HD2, KDAC2, RPD3, YAF1 |
| HDAC3 | histone deacetylase 3 | HD3, KDAC3, RPD3, RPD3-2 |
| HDAC8 | histone deacetylase 8 | CDA07, CDLS5, HD8, HDACL1, KDAC8, MRXS6, RPD3, WTS |
| TAF1 | TATA-box binding protein associated factor 1 | BA2R, CCG1, CCGS, DYT3, DYT3/TAF1, KAT4 |
| MLLT3 | MLLT3 super elongation complex subunit | AF9, YEATS3 |
| YEATS2 | YEATS domain containing 2 | FAME4 |
| KAT6A | lysine acetyltransferase 6A | ARTHS, MOZ, MRD32, MYST-3, MYST3, RUNXBP2, ZC2HC6A, ZNF220 |
| DPF2 | double PHD fingers 2 | CSS7, REQ, UBID4, ubi-d4 |


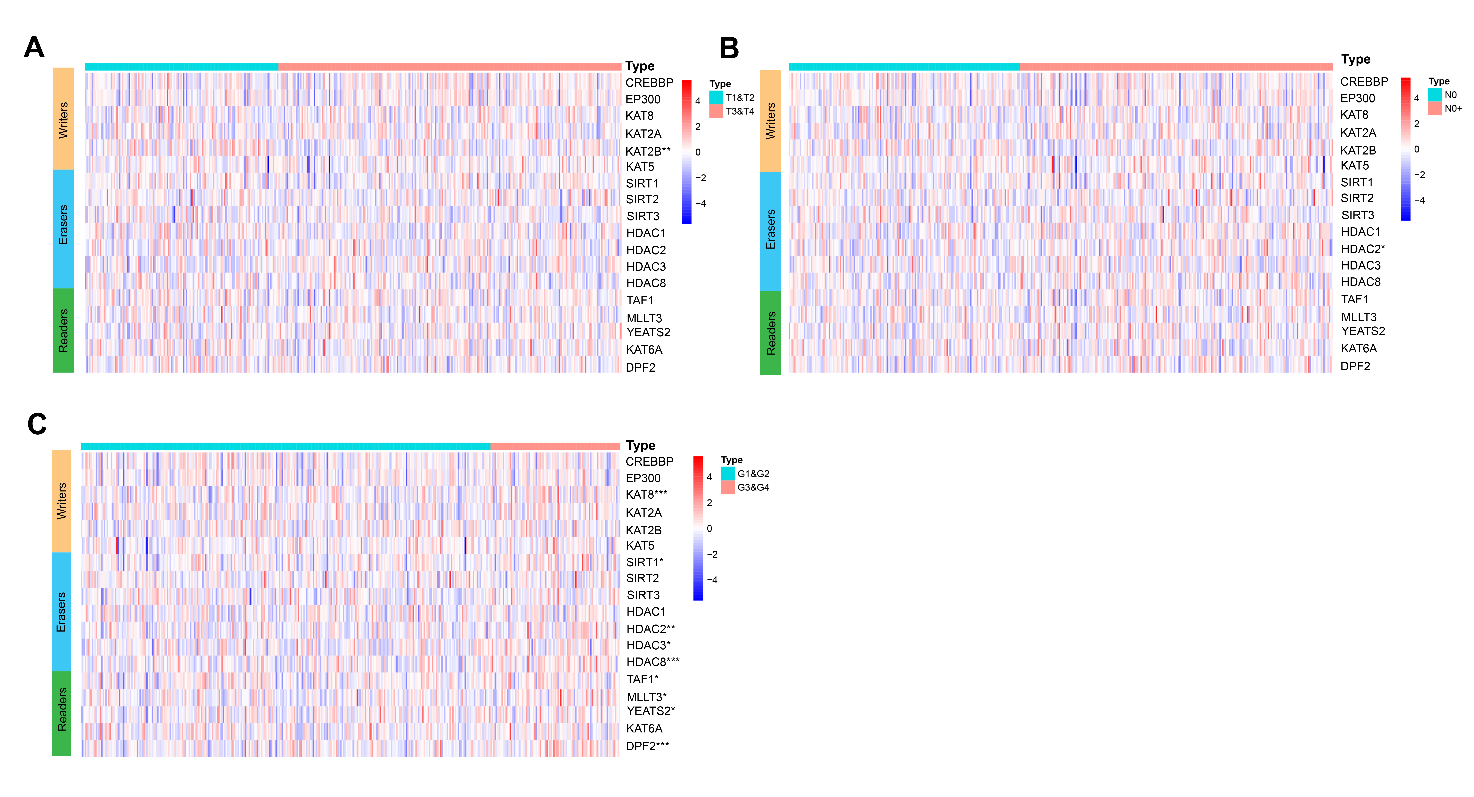


**Supplementary Figure 1. Expression of lysine crotonylation regulators in HNSCC with different clinicopathological features.** Expression levels of 18 lysine crotonylation regulators in HNSCC with (A) different T stage groups, (B) with or without lymph node metastasis, and (C) different histologic grades. * *p* < 0.05, ** *p* < 0.01, and *** *p* < 0.001.


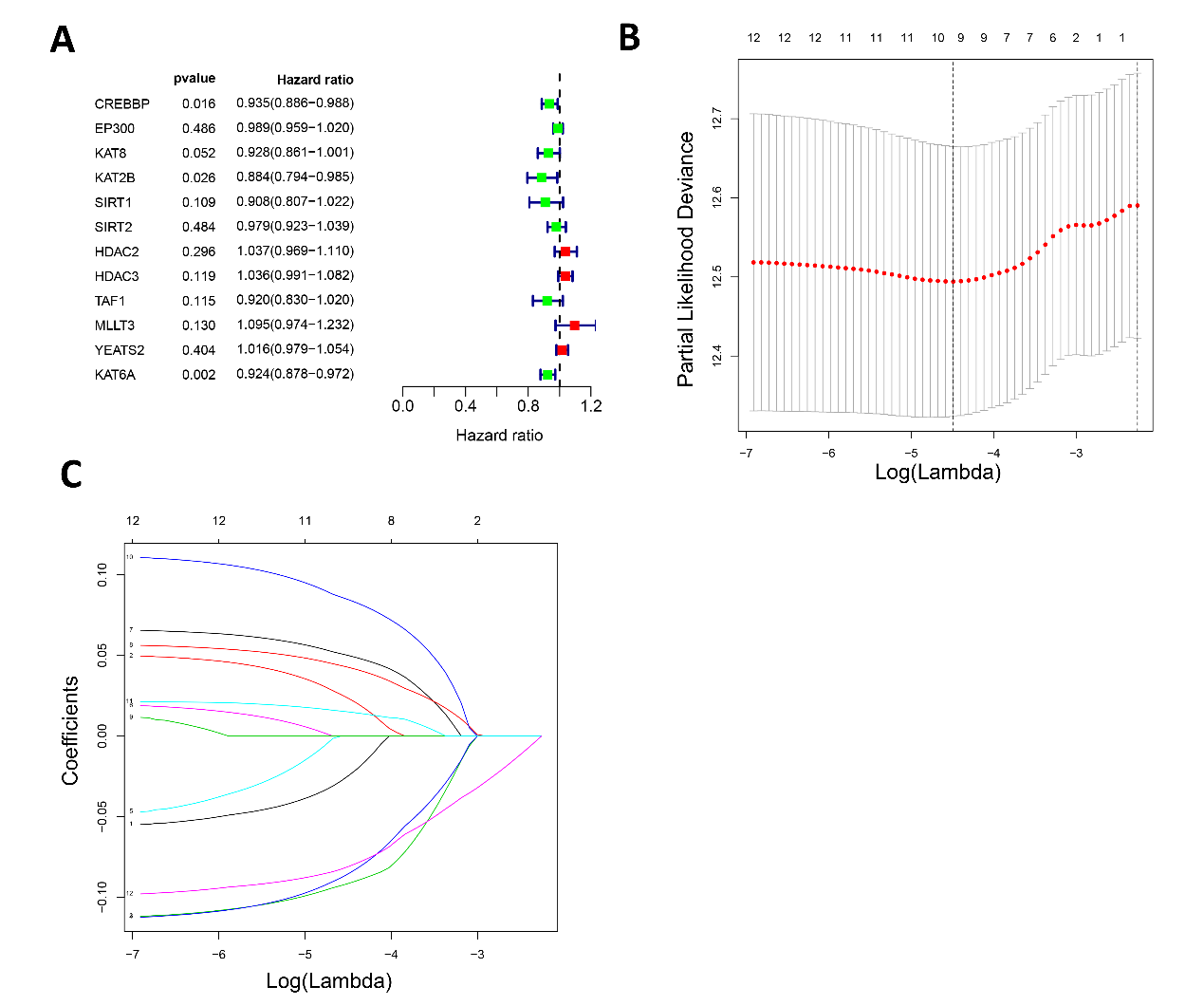


**Supplementary Figure 2**. **Construction of prognostic signatures based on the crotonylation regulators in TCGA-HNSCC.** (A) Univariate Cox regression of lysine crotonylation regulators on the prognosis of HNSCC patients in TCGA dataset; (B) The coefficient profile was plotted against the log (lambda) sequence. Selection of the optimal parameter (lambda) in the LASSO model for HNSCC; (C) LASSO coefficient profiles of the 18 crotonylation regulators in HNSCC.
